# Influence of Acrylonitrile Content on the Adhesive Properties of Water-Based Acrylic Pressure-Sensitive Adhesives

**DOI:** 10.3390/polym14050909

**Published:** 2022-02-24

**Authors:** Irene Márquez, Núria Paredes, Felipe Alarcia, José Ignacio Velasco

**Affiliations:** 1 Lubrizol Advanced Materials, Applications Department, Camí de Can Calders, 13, 08173 Sant Cugat del Vallès, Spain; irene.marquez@lubrizol.com (I.M.); nuria.paredes@lubrizol.com (N.P.); 2 Poly2 Group, Department of Materials Science and Engineering, Universitat Politècnica de Catalunya (UPC BarcelonaTech), ESEIAAT, Carrer de Colom, 11, 08222 Terrassa, Spain; felipealarcia@hotmail.com

**Keywords:** acrylic pressure-sensitive adhesives, emulsion polymerization, acrylonitrile

## Abstract

A series of pressure-sensitive adhesives (PSA) were prepared by emulsion polymerization in order to obtain a PSA that meet with the current label market requirements. For it, the effect of the incorporation of acrylonitrile (ACN) as hard monomer was investigated in a *n*-butyl acrylate (*n*-BA) and acrylic acid (AA) system. Great differences were found in the adhesive performance according to the ACN weight ratio. Its increased resulted in a considerable rise in the average sol molecular weight and in the glass transition temperature. This was reflected in a decrease of adhesion forces (peel resistance and tack) and an increase of the cohesion forces (shear resistance). Moreover, the incorporation of the minimum amount of ACN studied showed a great change in the elastic modulus determined by dynamic shear resistance with respect to the based formulation that did not contain ACN. Finally, the ice bucket test was carried out to check the adhesive performance in cold and wet environments.

## 1. Introduction

Acrylic pressure-sensitive adhesives (PSA) have wide market in fields such as tapes, labels or protective films because of their excellent adhesive properties, resistance to UV light and a good aging performance, among others [1]. They are obtained by free radical addition polymerization of alkyl esters of acrylic acid. These monomers can be classified into functionals, soft and hard monomers. The functional monomers are strong hydrogen bond donors which provide of reactive groups in the final polymer. Moderate polar monomers may be used up to about 30 wt % and highly polar monomers to about 10 wt %. Acrylic acid is the most widely used and studied functional monomer followed by methacrylic acid, itaconic acid or acrylamide. In a typical formulation, the soft monomers constitute from about 50–90 wt %. They have a high molecular weight and a low homopolymer glass transition temperature (T_g_) to ensure a soft and tacky film. However, useful PSA are not homopolymers since, in addition of tacky, they need a good balance between adhesion and cohesion forces needed to resist an applied shear stress. To provide cohesion to the PSA according to the demands of the application, a comonomer is usually added to increase the elastic modulus and T_g_. The homopolymer of these hard monomers have T_g_ greater than 30 °C, such as methacrylates, styrene, acrylonitrile (ACN), vinyl acetate, vinil chloride, vinylidene chloride, and butadiene [2,3].

One of the most challenges in the field of PSA is obtaining a good adhesion-cohesion balance. Adhesion is characterized by tack and peel resistance, whereas cohesion is characterized by shear resistance. Both are opposing forces and therefore when one increases, the other tends to decrease [4,5]. 

Different studies have been carried out to investigate the effect of hard monomers in the adhesive performance. Fang et al. [6] studied the use of different kinds of hard monomers in systems of *n*-butyl acrylate (*n*-BA), acrylic acid (AA) and 2-hydroxy ethyl acrylate (HEA). The effect of styrene (St), methyl methacrylate (MMA) and 2-phenoxy ethyl methacrylate (SR-340) monomers on the adhesive properties were studied. With the increase of hard monomer content, the gel content decreased and the T_g_ increased. In the case of St, the peel resistance increased up to a maximum, but beyond it, the PSA become too stiff, and the peel decreased. On the other hand, the increase of MMA and SR-340 decreased the peel resistance. However, SR-340 showed the lowest peel resistance among the three kinds of hard monomers because it showed the highest gel content of the latex. Xu et al. [7] synthetized a series of PSA with different MMA contents (0–30%) as hard monomer in a system of *n*-BA and AA. They found that the average sol molecular weight and the glass transition temperature (T_g_) linearly increased with MMA content. The shear resistance increased with the MMA but the peel resistance and tack decreased. This effect was attributed to the higher stiffness of the chains. On the other hand, some studies related to the effect of hard monomers and its polarity with the adhesive performance. Peykova [8] and Chan [9] observed in their respective studies that the incorporation of a small amount of polar comonomers such as ACN increased slightly the tack values of their adhesives.

**Figure 1:**
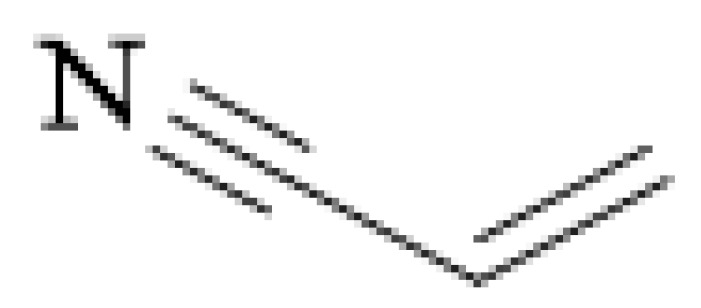
Acrylonitrile monomer structure.

ACN is one of most used hard monomers. It provides heat and chemical resistance to the final product [10]. This monomer is highly water soluble, 8.3 g/100 cm^3^ at 25 °C, and is more volatile than water. However, it become insoluble when polymerize due to the formation of strong polymer-polymer H bonds [11]. Moreover, it is considered a hard monomer due to the high T_g_ of its homopolymer, 105 °C [12]. As shown the Figure 1, the ACN has both the nitrile group and a double bond in the same molecule, which makes this monomer highly reactive and, for this reason, it must be inhibited on storage against polymerization [12,13].

However, very few references were found on its effect in acrylic-based PSA systems. Reddy et al. [14] used different ratios of ACN in the synthesis of pressure-sensitive adhesives (PSA). According to their study, small amount of a functional monomer such as ACN increases hydrophilicity and creates cross-linking sites which promote adhesion of the PSA with its increased moisture permeability. They observed that the PSA with increasing of ACN content exhibited higher T_g_ and higher peel and shear resistance values. Moreover, the ACN fraction in a mixture play an important role in the degree conversion. A higher fraction of ACN trends to produce a higher conversion in the polymerization. Zhang et al. [15] studied conversion rate for various weight ratios systems of ACN/*n*-BA (8/2, 7/3 and 6/4). They observed that the greater the fraction of ACN, the higher was the conversion rate. At 30 min, the conversion was of 81, 77 and 61 wt %, respectively. Capek et al. [16] studied the composition of ACN and *n*-BA copolymer and saw that when the higher monomer concentration of butyl acrylate in the oil phase due to the high water solubility of ACN give rise to the formation of polymer rich in butyl acrylate. Due to the large amount of ACN dissolved in water, the ACN content in polymer particles decreases and tends towards a very low value as soon as BA is completely depleted. The presence of butyl acrylate reduces its concentration in the aqueous phase. Butyl acrylate serves as an extraction agent to remove ACN from the water phase because the latter monomer is miscible with butyl acrylate. 

The general objective of the present study was to develop a PSA for glass bottle labels that met the current requirements of the wine cellars. For this, the adhesives must show values of peel resistance, tack and shear resistance close to 10 N/25 mm, 10 N and 24 h, respectively. Moreover, the adhesive must be able to withstand wet and cold environments.

In our previous article [17], the influence of the soft monomer in the final adhesive properties was studied. As the 2-ethylhexyl acrylate (2-EHA) proportion increased with respect the *n*-BA proportion, the peel resistance and tack values decreased. Instead, the shear values increased due to the increase in molecular weight. However, it was not possible to obtain a good balance between the three adhesive properties to meet current market requirements. For it, in this article, the effect of incorporating a hard monomer such as ACN monomer was studied. The same standard PSA formula based in *n*-BA and AA monomers was used as starting point. The ACN weight ratio was varying between 0 to 10 phm, partially replacing the *n*-BA monomer. 

The adhesives prepared were characterized by Fourier Transformed Infrared Spectroscopy (FTIR) and the T_g_ values were obtained by Differential Scanning Calorimetry (DSC). The gel content and the average sol molecular weight were determined by soxhlet extraction and Gel Permeation Chromatography (GPC). The adhesive properties, such peel resistance, tack and static shear resistance were investigated to determine if the adhesives synthetized met with the requirements established by the wine cellars. The dynamic shear resistance test allowed to show the viscoelastic properties of the adhesives produced. Finally, the ice bucket test was carried out to determine if the labels formed with these adhesives would be able to withstand wet and cold environments.

## 2. Materials and Methods

Deionized water was used in all reactions. Monomers *n*-butyl acrylate (*n*-BA, BASF, Ludwigshafen, Germany), acrylic acid (AA, BASF, Ludwigshafen, Germany) and acrylonitrile (ACN, IMCD Benelux B.V., Amsterdam, Netherlands) were used as received without further purification. As initiator was used ammonium peroxide sulphate from United Initiators (Pullach, Germany). Ammonium carbonate from BASF (Ludwigshafen, Germany) was used as buffer. The chain transfer agent used was tert-dodecyl mercaptan. The surfactant used was Dowfax^TM^ 2A1 from Dow Chemical (Midland, Texas, USA). In the redox system to decrease the residual free monomer the sodium formaldehyde sulfoxylate (Bruggolite^®^ E01) provided by Brüggemann KG (Heilbronn, Germany) was used as reducing agent and tert-butyl hydroperoxide (TBHP) from Pergan (Bocholt, Germany) was used as oxidizing agent. Solution of ammonia at 25 wt % was used to adjust the pH of the samples. The solvent used to extract the gel content and to dissolve the soluble part of the polymer samples was Tetrahydrofuran (THF, analytical grade from Merk, Hohenbrunn, Germany).

The substrates used for the adhesion tests were the Tintoretto qesso ultraWS^TM^ paper from Arconvert (Sant Gregori, Spain) and polyethylene terephthalate (PET) from Polinas (Manisa, Turkey). To build paper labels like those found on the market, silicone-coated paper (Gascogne Flexible, Dax, France) was used as release. NC 386 CYAN BASE FE gravure (Siegwerk, Barcelona, Spain) was used as ink. Doresco^®^ VMS7331 (Lubrizol, Sant Cugat, Spain) was used as coating. Aluminum (Umicore, Balzers, Liech-tenstein) was used to be metallized.

### 2.1. EmulsionPolymerization

By a semi-batch process 2 Kg of each latex were prepared in a 2.5 L glass reactor with mechanical stirring (100 rpm). All samples were adjusted at 55 wt % of solid content. The reactor composed by the 50% of the total deionized water, 0.1 phm of Dowfax^TM^ 2A1 and 3 phm of ammonium carbonate, was heated at 82 °C and purged with N_2_. After adding the initiator, the pre-emulsion feed consisting of the other 50% of deionized water, monomers (Table 1), 1.2 phm of Dowfax^TM^ 2A1 and 0.1 phm of chain transfer agent was added during 3 h at a constant rate.

To reduce the free residual monomer below 750 ppm, 2.0 phm of initiator agent was added (in two shots of 0.1 phm each one) and held for 1 h. In addition, once the reactor was cooled to 57 °C, 0.2 phm of TBHP and 0.3 phm of Bruggolite^®^ E01 were added as redox system and held for 4 h.

### 2.2. Latex Characterization

Once the latex was cooled at room temperature, it was filtered through a 150 µm filter. All the synthetized adhesives were adjusted to pH 7.5 with ammonia solution (12.5%) and to 50 wt % of solid content adding deionized water, as necessary.

By dynamic light scattering, the average particle size (PS) was measured with the Zetasizer Nano Series instrument at 25 °C using a detector with a 90° angle. The samples were prepared by diluting the latex in deionized water. The latex viscosity was determined at 25 °C using a programmable Brookfield DV-II+ Pro rotational viscometer.

The T_g_ was determined by differential scanning calorimetry (DSC) using the equipment DSC 1, STARe. Standards of indium and zinc were used to calibrate the equipment. The scanning cycles consisted of first heating from 25 °C to 200 °C at 20 °C/min and cooling from 200 °C to −65 °C at 20 °C/min and finally, a second heating from −65 °C to 200 °C, which was used to determine the T_g_ value.

The gel content, the insoluble polymer fraction in THF, was determined by Soxhlet extraction using THF as solvent at 70 °C for 24 h [18,19]. Once the extraction was done, the insoluble fraction was dried in an oven at 60 °C for 24 h to determine the gel content using Equation (1). W_1_ mean the initial weight of the filter, W_2_ is the filter with the dry polymer, and W_3_ is the filter weight with the dry polymer after extraction [20].

(1) $$\text{Gel}\;\text{content}\;(\%)\;=\;\frac{W_{3}-W_{1}}{W_{2}-W_{1}}\cdot 100$$

The polymer fraction soluble in THF was used to determine average molecular weight (M_w_) by GPC. These soluble fractions were dried in an oven at 60 °C until constant weight to be subsequently resolved in THF to achieve a concentration of about 0.01 g/mL. 10 μL of each sample were injected into the GPC instrument composet by a Waters 2414 refractive index detector and a Waters e2695 Separation Module. The last one was equipped with two lineal column, a Agilent PL Gel Mixed-C of 5 µm of 7.8 × 300 mm followed by a Styragel HR5E of 5 µm of 7.8 × 300 mm, and with one monopore column of Styragel HR 4 7.8 × 300 mm. The analisis was carried out at 40 °C using a THF flow rate of 1 mL/min. Various standards of polystyrene were used to calibrate the equipment. The average molecular weight was referred to them.

### 2.3. Adhesion Tests

The adhesive properties were determined through peel resistance, tack and shear resistance (static and dynamic) tests. 50 g/m^2^ of latex were cast were applied onto silicone-coated paper and dried for 1 min at 100 °C. The adhesive layer was subsequently transferred to the corresponding substrate. Five samples per latex were performed in each one of the different tests.

The peel resistance was evaluated at 180° angle at a speed of 300 mm/min with a Zwick/Roell Z 2.5 tensioner 24 h later to apply the tapes of both substrates (275 × 25 mm^2^) onto glass supports. The reported values are the average force and the failure type obtained during the test [21].

The loop tack test was carried out with an AT1000 tensile tester. Tapes of both substrates (175 × 25 mm^2^) forming a loop were deposited onto the study support (glass panel) in a controlled manner at a constant speed of 300 mm/min. The reported values correspond to the maximum force required to peel off the tapes and the failure type observed during the test [22,23].

The static shear test was carried out applying tapes of both substrates (25 × 25 mm^2^) onto stainless-steel panels holding 1 kg until failure. The average time to shear the tape from the test panel was recorded [24].

The dynamic shear resistance test was carried out at 5 mm/min with a Zwick/Roell Z 2.5 machine. PET tapes of 2.5 × 2.5 mm^2^ were adhered on untreated steel panels 20 min before the test [25]. The shear stress versus strain curves were recorded. The elastic modulus (G), the maximum stress (τ_m_) values, and the deformation energy up to failure (U) were determined from these curves.

The shear modulus was determined as the initial slope of the curve with a linear correlation coefficient (r^2^) being higher than 0.999 in all cases. The shear strength was determined as the maximum stress value in the test, and the deformation energy was calculated as the area under the curve up to the maximum stress value.

### 2.4. Water Resistance Tests

The water resistance was evaluated by the ice bucket test [26]. For it, five labels per latex were performed. 25 g/m^2^ of dry adhesive were transferred of silicone-coated paper to the paper substrate with a multilayer barrier coating to simulate the commercial labels. It consisted of a coating of Doresco^®^ VMS7331 (2.4 g/m^2^) followed by an aluminum layer of 0.035 μm metallized by vacuum deposition. To protect this layer 1.2 g/m^2^ of Doresco^®^ VMS7331 were applied followed by a gravure ink. Once the label was built, it was placed in a glass bottle and 24 h later it was submerged in an ice-water bath (1:1). After 24 h, the label integrity was check when the bottle was removed from the bath. 

## 3. Results and Discussion

### 3.1. Latex Phisico-Chemical Properties

The average particle size and viscosity of the aqueous polymer solution (50% solids) as well as the T_g_, gel content, and average sol molecular weight (M_w_) of the polymer are summarized in Table 2.

The introduction of 2 phm of ACN monomer in the formulation caused a slight increase in particle size, which was reflected in a certain decrease in viscosity. However, from 4 phm of ACN, the particle size and viscosity values were very similar. On the other hand, unlike the samples prepared in the previous article [17], with 2-EHA monomer, in this case the gel contents generated in these samples were negligible in all cases. Possibly because the lateral group of 2-EHA is bulkier than the nitrile group, which produced greater steric hindering and molecular entanglement. Moreover, it should be noted that the same amount of chain transfer agent was used in both studies. This chain transfer agent promotes the polymer chain terminations and formation of new chains, thus resulting in low molecular weight [27,28,29].

Although with the increase of this monomer no significant variations in gel content were obtained, a large increase in the average molecular weight was observed. With only 10 phm, the average molecular weight was tripled with respect to the sample E0. The propagation rate constant of ACN is an order of magnitude greater than that of *n*-butyl acrylate (28,000 and 2100 L/mol·s, respectively [30]). Therefore, with increasing ACN content, the polymerization rate increased, and this was reflected in an increase in molecular weight. 

**Figure 2:**
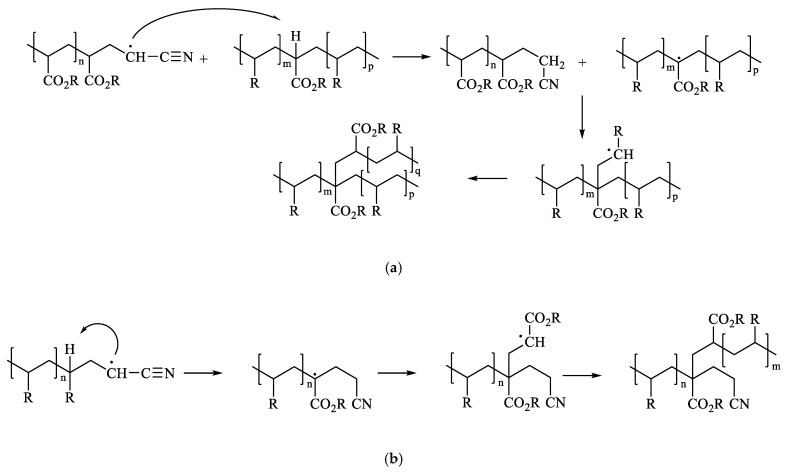
Mechanism proposed by transfer to polymer reactions: (**a**) Intermolecular chain transfer to polymer. (**b**) Intramolecular chain transfer to polymer (backbiting).

Moreover, ACN, as the AA and *n*-BA, can form branch points by intermolecular chain transfer to polymer followed by termination by combination and by intramolecular chain transfer (backbiting) [31,32,33]. According to McCord et al. [34], terminal ACN groups are formed by H-abstraction. Figure 2 shows the possible mechanisms that may take place to account for this increase in molecular weight when incorporating the monomer ACN.

**Figure 3:**
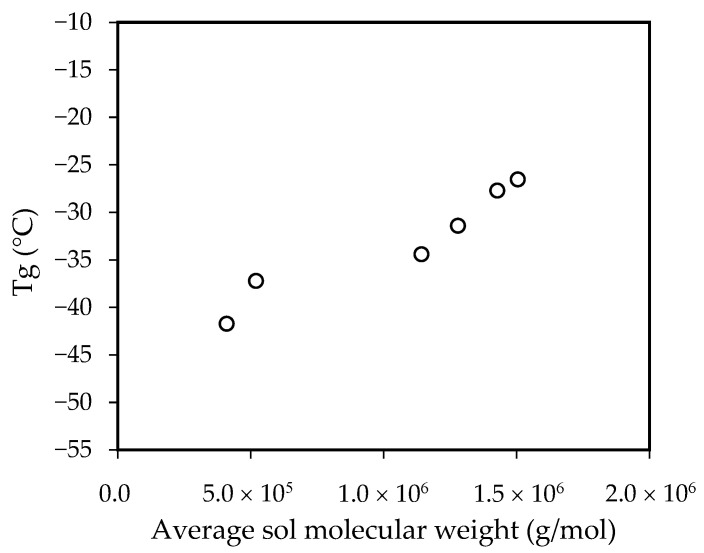
Influence of average sol molecular weight in the T_g_ values.

It was reflected in the T_g_ values as shown Figure 3.

From the sample E0 to the sample N10, an increase of 15 °C in the T_g_ value was observed. According to the literature, the T_g_ of the ACN homopolymer is 105 °C [12]. Clearly, a small amount of this monomer was enough to have a great effect on T_g_ values since its T_g_ is much higher than that of *n*-BA (−54 °C [35]).

**Figure 4:**
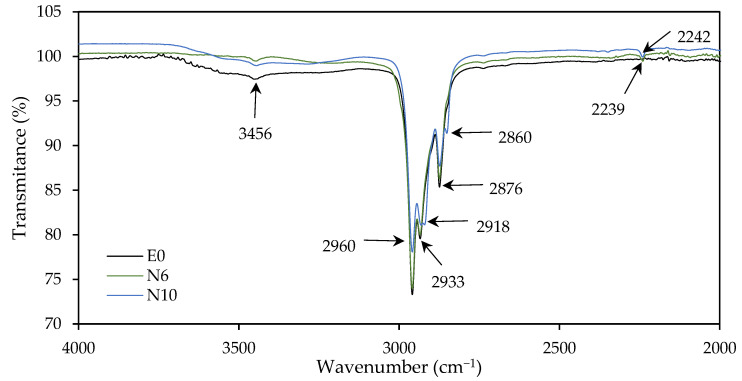
FTIR spectra of E0, N6 and N10 samples over a range of wavenumber of 4000−2000 cm^−1^.

**Figure 5:**
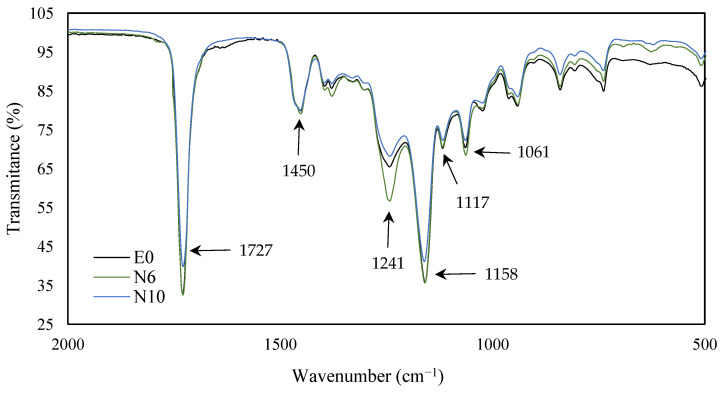
FTIR spectra of E0, N6 and N10 samples over a range of wavenumber of 2000−500 cm^−1^.

To see how the incorporation of this comonomer affected the structure of the adhesive, the samples were analyzed by FTIR. Figure 4 and Figure 5 show changes in some characteristic bands according to the weight ratio ACN/*n*-BA.

The broad band in the range 3100–2700 cm^−1^ with its maximum at 2960 cm^−1^ was assigned to C–H stretching in CH, CH_2_, and CH_3_. The bands at 2960, 2933, 2918 and 2876 cm^−1^ decrease their intensity with the increase of ACN monomer. Moreover, with the increase of this monomer, the singlet at 2876 and 2933 cm^−1^ become in a doublet with the appearance of another band at 2860 and 2918 cm^−1^, respectively.

The band at 2240 cm^−1^ appeared with the presence of ACN, corresponding to the nitrile group (C≡N). The strong band at 1730–1720 cm^−1^, characteristic from acrylic polymers, correspond to the C=O stretching of ester group. With the increase of ACN group, a longer distance of the acryl group (R–C=O) from the backbone was reflected in the IR spectra with the reduction of peak at 1728 cm^−1^ [36,37]. The peaks at 1241 and 1158 cm^−1^ were due to the C–O stretching vibration of the ester group. With the increase of ACN, the bands at 1117 and 1061, corresponding to C–C–C deformation of tertiary carbon, were reduced their intensity [38].

### 3.2. Adhesive Properties

The adhesive properties (tack, peel resistance and static shear resistance) of polymers, tested on paper and PET tapes, were measured and summarized in Table 3.

With both substrates the same effect was observed but the differences were greater in the case of the paper substrate. As paper is a porous substrate, the adhesive penetrated the matrix resulting on a higher anchorage, i.e., the interfacial adhesive-substrate strength was higher than in the case of PET substrate [39]. 

On the other hand, also keep in mind that the deformation of the paper that takes place during the test is part of the fracture energy of the process. However, thus does not happen with PET because it is an elastic substrate.

On paper substrate, both peel resistance and tack decreased with increasing proportion of ACN. As is known, it is influenced by the chains of lower molecular weight and the peel by those of the middle-range molecular weight. By introducing a hard monomer, the molecular weight was increased, thus decreasing the chains of middle and low-range molecular weight. 

On paper substrate, the N10 adhesive, with only 10 phm of ACN, showed peel resistance and tack values 7 and 9 times respectively lowers than E0. On PET substrate, the difference in the peel resistance and tack values were not as marked. The N10 adhesive showed in both tests, values 2 times lower than E0. On both substrates, the adhesive E0 and N2 showed a cohesive failure in the peel resistance test since the adhesive-substrate interfacial strength was higher than the strength of the adhesive itself. Nevertheless, the rest of adhesives showed an adhesive failure due to the increased molecular weight of the adhesive chains. 

As the degree of entanglement and crosslinking increased, the cohesive strength of the adhesive increased, thus decreasing the adhesive-substrate interfacial strength. This increase in the cohesion of the adhesive when increasing the weight-ratio of ACN was reflected in an increase of the static shear resistance values. The adhesive N10 increased almost 179 times in the paper tapes and 22 times in the PET tapes respect E0. 

The most significant changes in tack, peel and shear resistance values were observed between samples E0-N2 and N2-N4. Instead, the differences between the rest of samples (N6, N8 and N10) when also increasing 2 phm of ACN between them, were smaller. On paper substrate, if the differences between E0 and N2 are compared, N2 adhesive showed a decrease of 7 units in peel resistance and of 13 units in tack values and an increase of 5 units in shear resistance. However, if the differences between N6 and N8 are compared, N8 adhesive showed a decrease in the peel resistance and tack values of 3 units and 2 units, respectively, and an increase in shear of 2 units respect N6.

If the results obtained in this section are compared with the results of adhesion obtained in the previous article [17], clearly ACN monomer has a greater effect on adhesive properties than 2-ethylhexyl acrylate monomer. 

The incorporation of both monomers in the standard formulation, considerably decreased the peel and tack values respect E0. The minimum amount studied of 2-EHA was 14.6 phm (E15) and the maximum amount of ACN studied was 10 phm (N10). If both adhesives are compared, in the case of the paper substrate, the N10 adhesive, showed a decrease of peel resistance and tack of 85% and 89% respectively. However, E15 only showed a decrease of 4% and 11%, respectively.

**Figure 6:**
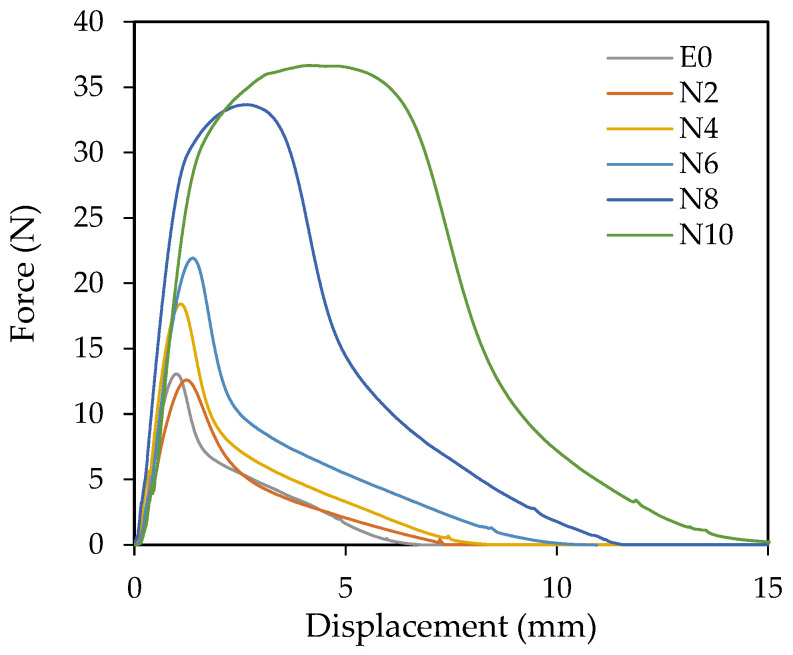
Representative force-displacement curves obtained by dynamic shear test.

On the other hand, the dynamic shear resistance was evaluated to obtain more information about the material resistance to a shear stress applied. Figure 6 shows the force-displacement curves obtained in the test.

The curves show the typical behavior of a dynamic shear test [40], with a linear initial zone, which allows to determine the elastic modulus, a maximum stress that defines the strength of the adhesive related to its internal cohesion, and a deformation up to failure that is a measure of its ductility. The area under these curves determines their strain energy or toughness. With increasing ACN content, both the stress and the maximum strain of the curves increased. The adhesives N8 and N10, which were formulated with the highest amounts of ACN, showed a considerably higher toughness than the rest of samples.

**Figure 7:**
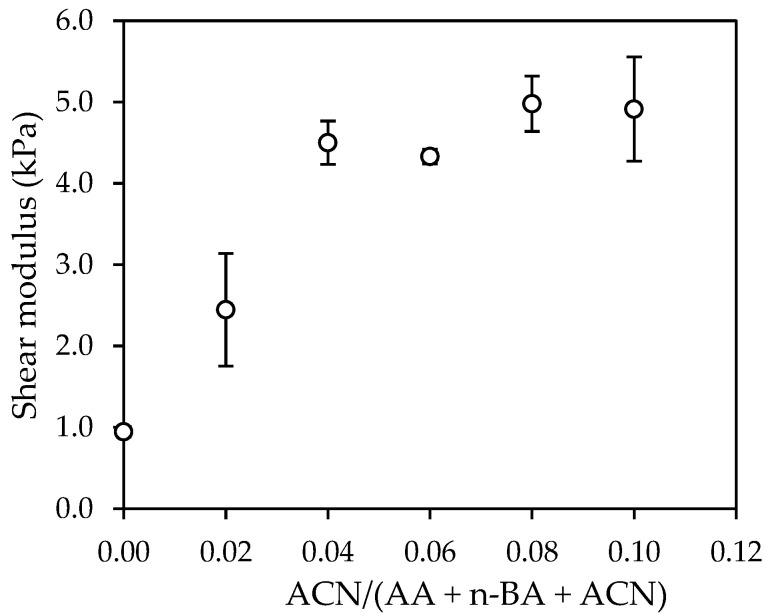
Effect of acrylonitrile content on the elastic modulus.

The elastic shear modulus (G) increased with the ACN content (Figure 7), as expected from the increase in the polymer T_g_.

The most significant variations were found between the samples E0, N2 and N4 as previously observed in the adhesive properties. Samples N2 and N4 showed an increase of G of almost 2.5 and 5 times, respectively, with regard to E0.

**Figure 8:**
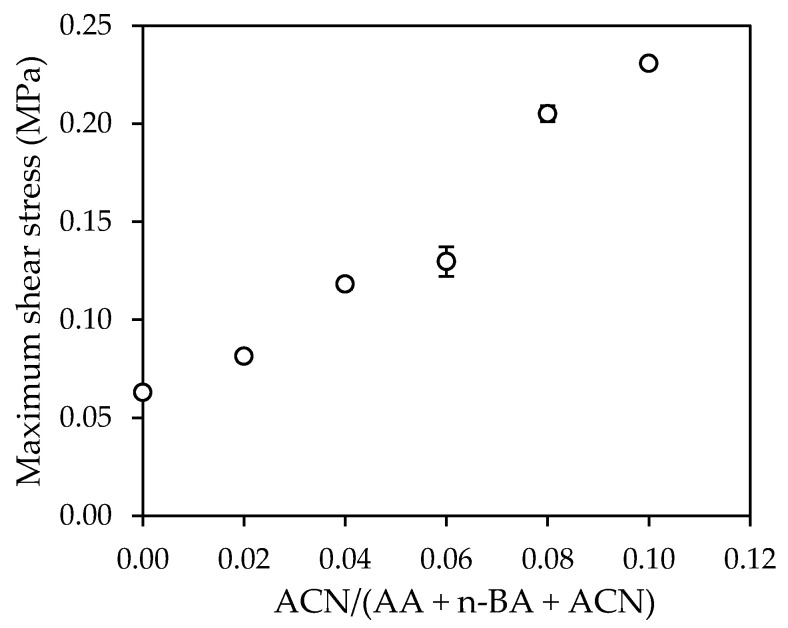
Effect of acrylonitrile content on the maximum shear stress.

The maximum shear stress recorded in the dynamic shear test is represented in Figure 8.

Also in this case, it is observed an increase in this property with the ACN content, as a consequence of increased molecular weight.

**Figure 9:**
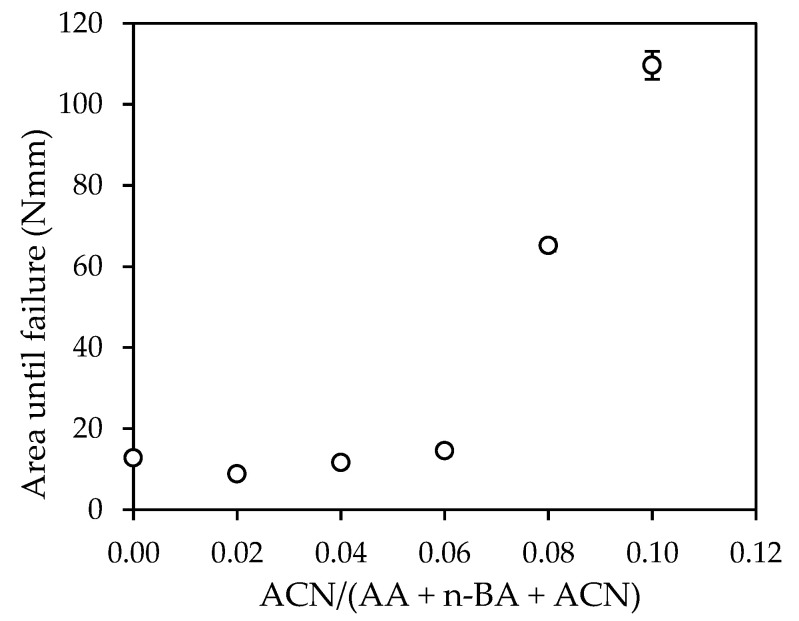
Effect of acrylonitrile content on the deformation energy until failure of the adhesive.

Finally, the toughness of the adhesives was determined as the deformation energy up to the adhesive failure in the dynamic shear test and was calculated from the area under the curve force-displacement up to the maximum force value as shown in Figure 9.

Samples N8 and N10 required more energy than the rest. N8 and N10 needed 5 and 9 times, respectively, more energy than E0. However, the rest of the samples showed very similar energy values between them.

### 3.3. Water Resistance

**Figure 10:**
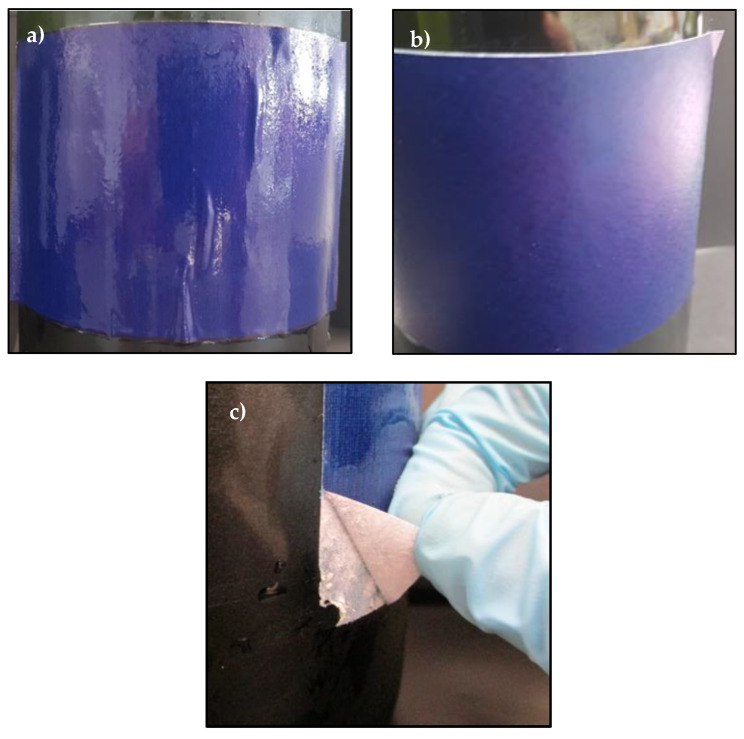
Optical pictures of the different states of a label of a same bottle using the adhesive N6 in the ice bucket test: (**a**) label just after remove the bottle from the ice bucket, (**b**) dried label after 24 h to remove the bottle from the ice bucket and (**c**) structural failure upon peel off the label manually.

Finally, to determine the water resistance of the adhesive in wet and cold conditions, the ice bucket test was carried out. All labels behaved in the same way. Figure 10 shown as example the behavior of the label built with the adhesive N6.

After 24 h submerged in the water:ice (1:1 by weight) bath, the labels remained stuck but with bubbles and wrinkles on the edges of the label as shown in Figure 10a. On the other hand, 24 h after the bottle was taken from the ice bucket, the bubbles and wrinkles disappeared but the ends of the label were slightly peeled off as shown in Figure 10b. Finally, when the label was removed from the bottle surface manually, structural failures occurred as shown in Figure 10c.

## 4. Conclusions

In order to obtain a PSA that meets the adhesive properties required by wineries, the influence of the incorporate in the formulation a hard monomer such as ACN was investigated. A series of acrylic PSA were prepared by emulsion polymerization with different weight-ratios of ACN on a system composed by acrylic acid and *n*-butyl acrylate. 

The results did not show significant changes in particle size and viscosity. However, a slight increase in particle size was observed if the adhesive E0 is compared with N2, when 2 phm of ACN were introduced in the adhesive composition. This was also reflected in a slight decrease in viscosity. On the other hand, the gel content generated in these samples was negligible in all cases. However, great increase in the average sol molecular weight were observed by GPC with the increase of ACN. This was also reflected in an increase in T_g_.

Regarding to the adhesive properties, on both substrates, the results showed the same trend. With the increase in the amount of ACN, the molecular weight increased and therefore the peel resistance and tack values decreased. However, this greatly favored by the static shear results. The most marked changes in tack, peel and shear resistance values were observed between samples E0-N2 and N2-N4 on paper substrate. Considering the values required of peel resistance, tack and shear resistance by the wine industries were 10 N/25 mm, 10 N and 24 h respectively, on both substrates, the adhesive properties of N4 (7.5 ± 0.6 N/25 mm, 9.0 ± 0.2 N and 15.2 h, respectively) and N6 (6.7 ± 0.3 N/25 mm, 5.0 ± 0.9 N and 16.4 h, respectively) were the closet.

The force-displacement diagrams obtained in the dynamic shear test showed that the adhesives N8 and N10, which were formulated with the highest amounts of ACN, showed a considerably higher toughness than the rest of samples. The shear elastic modulus (G) obtained for the samples N2 and N4 was almost 3 and 5 times, respectively, higher than E0. The maximum shear stress increased in a constant way with the increase in the ACN content. Samples N8 and N10 required 5 and 9 times, respectively, more deformation energy than E0. The rest of adhesives showed a deformation energy very similar to E0.

In the ice bucket test, the labels remain stuck all time, but they showed bubbles and wrinkles on the edges. When the labels were dried, the bubbles and wrinkles disappeared. However, the ends of the labels were slightly peeled off. This is an inconvenient for the end user as the label is expected to always remain in perfect condition. Therefore, it will be necessary to improve the water resistance of these adhesives.

## Figures and Tables

**Table 1:** Monomer composition (phm) of the adhesives studied.

Sample	*n*-BA	ACN	AA
E0	97.5	0.0	2.5
N2	95.5	2.0	2.5
N4	93.5	4.0	2.5
N6	91.5	6.0	2.5
N8	89.5	8.0	2.5
N10	87.5	10.0	2.5

**Table 2:** Physico-chemical properties.

Sample	Particle Size (nm)	Viscosity (cp)	T_g_ (°C)	Gel Content (%)	M_w_ (g/mol)
E0	189	183	−41.7	2.3	410,224
N2	198	150	−37.2	2.0	520,650
N4	224	79	−34.4	5.9	1,143,185
N6	227	70	−31.4	6.4	1,280,423
N8	228	73	−27.7	5.7	1,428,458
N10	222	75	−26.6	5.8	1,505,040

**Table 3:** Adhesive properties determined on paper and PET tapes.

	Paper Tape			PET Tape		
	Peel Resistance (N/25 mm)	Tack (N)	Shear Resistance (h)	Peel Resistance (N/25 mm)	Tack (N)	Shear Resistance (h)
E0	28.1 ± 0.5	26.0 ± 0.9	0.1 ± 0.0	13.6 ± 0.2	8.6 ± 1.0	1.8 ± 0.1
N2	21.2 ± 0.7	12.5 ± 0.9	5.6 ± 0.9	10.6 ± 0.3	8.0 ± 0.3	4.0 ± 0.3
N4	7.5 ± 0.6	9.0 ± 0.2	15.2 ± 3.4	8.3 ± 0.1	7.3 ± 1.0	14.5 ± 0.7
N6	6.7 ± 0.3	5.0 ± 0.9	16.4 ± 2.1	9.2 ± 0.3	4.7 ± 0.4	30.5 ± 3.1
N8	4.3 ± 0.4	3.1 ± 0.4	18.4 ± 2.9	8.4 ± 0.1	4.0 ± 0.5	36.3 ± 2.8
N10	4.2 ± 0.1	2.9 ± 0.4	21.5 ± 4.0	6.0 ± 0.3	3.9 ± 0.4	40.3 ± 3.0

## Data Availability

The data presented in this study are available on request from the corresponding author.
